# The role of China's acute seizure action plan in the management of out-of-hospital seizures in children with epilepsy: a pilot study

**DOI:** 10.3389/fpubh.2026.1707312

**Published:** 2026-03-16

**Authors:** Nan Zhang, Shuangzi Li, Ting Wang, Qing Xia, Cui Cui

**Affiliations:** 1 Department of Nursing, Children's Hospital of Chongqing Medical University, National Clinical Research Center for Children and Adolescents' Health and Diseases, Ministry of Education Key Laboratory of Child Development and Disorders, Chongqing Key Laboratory of Child Neurodevelopment and Cognitive Disorders, Chongqing, China; 2 Department of Neurology, Children's Hospital of Chongqing Medical University, National Clinical Research Center for Children and Adolescents' Health and Diseases, Ministry of Education Key Laboratory of Child Development and Disorders, Chongqing Key Laboratory of Child Neurodevelopment and Cognitive Disorders, Chongqing, China

**Keywords:** action plan, acute seizure, children, epilepsy, out-of-hospital seizures

## Abstract

**Introduction:**

This study aimed to explore the function and applicability of localized China acute seizure action plan (CASAP) in the management of out-of-hospital seizures in children with epilepsy, and to provide a theoretical basis and practical framework for further optimisation and promotion of this action plan.

**Methods:**

A self-paired research design was employed. The CASAP was used to provide out-of-hospital seizure management guidance to the primary family caregivers of children with epilepsy. The children were followed up for six months after the intervention.

**Results:**

Sixty two children aged 0–18 years participated. The M (P25, P75) of the total seizures in children with epilepsy within six months before intervention with CASAP was nine (3–169), which was lower than seven (0–20) within six months after intervention (*P* < 0.05). The M of the average duration of seizures before intervention was 1.5 (1.0–5.4), and 0.5 (0.0–1.0) after intervention (*P* < 0.001). After intervention, number of children with no emergency visits increased from 35 (56) to 47 (76%), and those with ≥1 emergency visit decreased from 27 (44) to 15 (24%); (*P* < 0.05).

**Discussion:**

The results provide beneficial insights for out-of-hospital seizure management and improves health outcomes in children.

## Introduction

Epilepsy is a complex, chronic brain dysfunction disorder with multiple etiologies and is a common neurological disorder in children (1). In 2016, the Global Burden of Disease (GBD) survey showed that the global prevalence of epilepsy ranged from 540.01 per 100,000 to 737.00 per 100,000 (2). From 1990 to 2019, the incidence rate of epilepsy among children and adolescents under 20 years of age in China rose from 24.59 per 100,000 to 36.64 per 100,000 and the prevalence rate increased from 181.14 per 100,000 to 230.99 per 100,000 (3). Currently, the number of patients with epilepsy in China exceeds 10 million, and approximately three-quarters of them have childhood onset (4, 5). Epileptic seizures affects children's physical, psychological, and social health and imposes a burden on individuals, families, and society (6, 7). Approximately one-third of patients are unable to effectively control epileptic seizures and develop acute convulsive seizures (AS) (8). AS is one of the most common neurological emergencies in children with epilepsy and can lead to death, complications, cognitive behavioral disorders, or epilepsy. Timely and effective out-of-hospital seizure management is crucial for the prognosis of children (9).

In 2003, the British health department proposed the “Action Plan for Improving Services for Epilepsy Patients” (10), aiming to address the insufficient management of epilepsy seizures outside hospitals. With the broad application of this action plan and the continuous optimisation of relevant guidelines and consensus on epileptic seizure management, acute seizure action plan (ASAP) has gradually become an action guideline and educational tool for neurospecialized medical staff to provide out-of-hospital epileptic seizure management for epileptic patients and their family members, and school medical staff (11). ASAP is essentially an individualized and written disease management tool. Its core function is to provide a step-by-step pre-hospital emergency intervention guidance when epileptic seizures or recurrent convulsions occur (12). In 2021, a consensus document of the American Epilepsy Foundation recommended ASAP as an important management tool for the out-of-hospital emergency treatment of children with ankylotic and clustered epileptic seizures (13).

Presently, the ASAP is extensively applied abroad and has improved the emergency management level of out-of-hospital seizures in children with epilepsy (14). The ASAP can be regarded as a milestone in the management of acute seizures in children with epilepsy (15). Standardized seizure management guidance can effectively empower family caregivers, teachers, and community workers to manage seizures outside the hospital in a more timely, safe, and standardized manner in nonmedical institutions, such as families, communities, and schools, and optimize the management effect of seizures outside the hospital. However, due to differences in cultural backgrounds, educational levels, and medical systems among different countries and regions, the ASAP tool may have adaptability issues in the process of cross-cultural application, affecting its acceptance and implementation. Therefore, when introducing and applying the ASAP tool, it is necessary to consider the importance of localization and build an ASAP tool that suits national conditions.

This study aimed to preliminarily explore and validate the efficacy of the China's Acute Seizure Action Plan (CASAP) in children with epilepsy based on authentic evidence and in line with domestic clinical contexts for the management of seizures in children with epilepsy outside the hospital, thereby providing scientific and effective intervention measures for the management of seizures in children with epilepsy outside the hospital.

## Methods

### Development of CASAP

**Figure 1:**
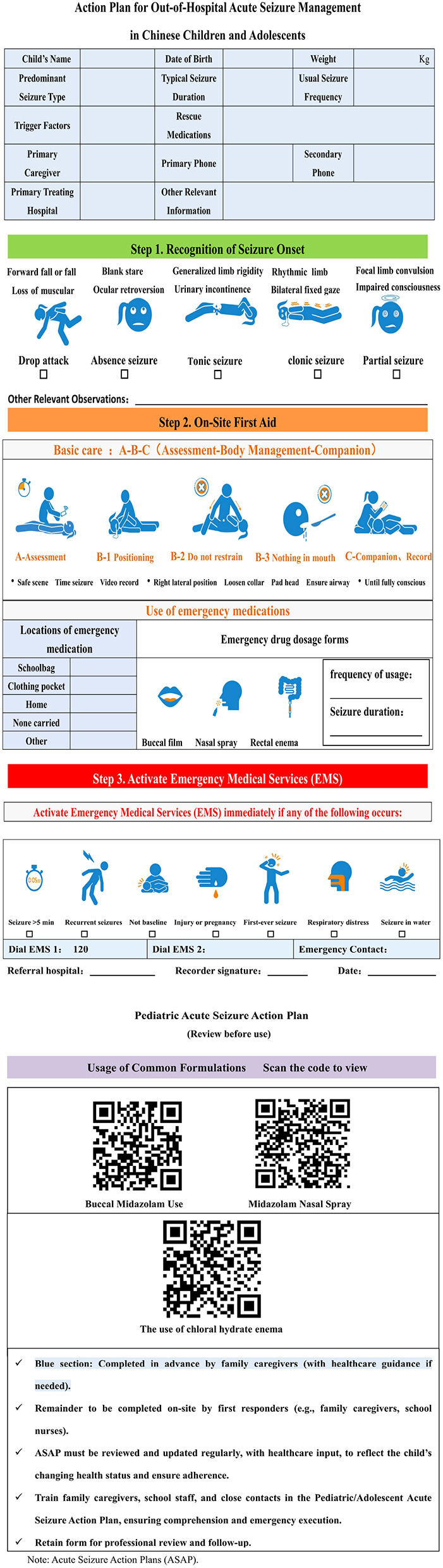
Action plan for out-of-hospital acute seizure management in Chinese children and adolescents.

Before initiating the ASAP tool, the cultural differences at home and abroad as well as the localization characteristics in China were considered. In the early stages of the study, our research team, based on a literature review (8) and relevant evidence (16) summary, combined with localized research on the knowledge, attitude, and practice of out-of-hospital seizures among Chinese children with epilepsy (17), and gained a comprehensive understanding of the current status of cognition, attitude, and behavior toward seizures among domestic children with epilepsy and their families. Using the evidence summary method, core points and key strategies for epileptic seizure management suitable for China's national conditions were identified (16). Subsequently, through Delphi expert enquiries, multiple rounds of evaluations and optimisations were conducted on the content, form, promotion channels, of the ASAP tool, thereby constructing a CASAP tool with Chinese characteristics. This CASAP tool has obtained the National Work Registration Certificate of China (Registration Number: Guozuodengzi−2024-L-00055068). The CASAP developed in this study is shown in Figure 1.

### Structure and core components of the CASAP

The CASAP is designed as a structured, user-friendly guide comprising three core sections to facilitate implementation and adherence. Section 1 collects individual and caregiver information, including essential demographic and clinical details of the child with epilepsy and the primary family caregivers, to personalize the plan and ensure accurate identification. Section 2 outlines the core seizure management protocol, which serves as the central component of the CASAP. It is subdivided into: (a) seizure symptom identification, with clear descriptions and visual aids to help caregivers recognize seizure types specific to their child; (b) step-by-step first aid procedures, providing evidence-based sequential instructions for safe on-site management during an active seizure; (c) guidelines for seeking emergency assistance, specifying criteria for medical emergencies and instructions for contacting emergency services. Section 3 covers the learning content that must be completed before using CASAP, including the methods of common drug preparations for acute epileptic attacks and instructions for using CASAP.

### Participant recruitment

Using convenience sampling, children with epilepsy who visited the Department of Neurology of the Children's Hospital Affiliated with Chongqing Medical University from April 2022 to April 2023 were selected as the research subjects of this study. The inclusion criteria were as follows: (1) Compliance with the Seizure and Classification of Epilepsy in 2017 by the International League Against Epilepsy, (ILAE); (2) age between 0 and 18 years; (3) provision of informed consent by the patient and his/her guardian/caregiver. The exclusion criteria were as follows: (1) the patient and his/her guardian/caregiver's refusal to participate; (2) the disease was in a critical stage; (3) treatment was interrupted or discontinued; (4) family caregivers were unable to manage or follow up on epileptic seizures due to limited cognitive function. Information on the included and excluded patients was verified through enquiries with the participants and by clinical nursing staff reviewing the electronic medical records.

### Program description

This study adopted a self-sequential paired research design. Before the large-scale promotion and application of the CASAP, the research team selected the neurology center of a tertiary children's hospital in the southwest region for a small-scale prospective pilot application study.

Based on regular health education, all family caregivers of children with epilepsy included in the study received an additional integrated and participatory CASAP promotion education program. The core of this program was the application of a diversified CASAP health education toolkit, and its specific implementation was led by trained pediatric neurology nurses. The CASAP health education toolkit consists mainly of a paper version of the CASAP and videos on the procedures for handling out-of-hospital seizures in children with epilepsy. The application and implementation of the toolkit were carried out by pediatric neurology nurses through structured, one-on-one health education sessions combined with skill demonstrations and supervised practice.

The initial education session was arranged at the time of first distribution the paper-based CASAP, typically during a regular neurology follow-up visit. The nurse explained each component of the CASAP item-by-item and demonstrated the management of out-of-hospital seizures using the instructional video. Each session lasted approximately 20–30 min to ensure comprehension and allow time for caregiver demonstration and practice. Caregiver competence was assessed immediately after this session using the study's core instrument, the CASAP. Assessment involved two components: (1) evaluating key procedural skills; (2) a structured verbal recall assessment to confirm understanding of critical information from the CASAP protocol. Competency was defined as successful independent demonstration of all skills on the checklist and correct verbal recall of ≥90% of the key points outlined in the CASAP.

To ensure sustained competence and address individual learning needs, documentation of competence required a minimum of 2. The exact number within this range was determined by the assessing nurse based on the initial competency assessment using the CASAP and the caregiver's learning progression. For caregivers who achieved competency initially, at least one follow-up reinforcement session was scheduled. For complex cases or those facing initial learning difficulties, additional follow-ups were provided. Follow-up occurred via planned phone calls or outpatient visits 1–2 weeks after the initial session to answer questions and consolidate core content. Additionally, a combination of online and offline methods was adopted to support long-term retention. CASAP-related health educational activities were conducted at least twice a month, including one scheduled outpatient follow-up and one online resource push.

### Data collection

The General Information questionnaire, designed by researchers, collected children's demographic data and medical history, including comorbidities, antiepileptic drug use, seizure frequency and duration, emergency visits, and EEG results. The Family APGAR Index questionnaire was employed to assess perceived family function and support (18). Scores ranged from 0 to 10, categorized as severe dysfunction (0–3), moderate dysfunction (4–6), and good function (7–10). Its use was justified for three key reasons: first, the successful out-of-hospital management of acute seizures in children with epilepsy is heavily dependent on the family's supportive functioning and adaptability. Second, the APGAR's brevity and focus on perceived support make it particularly suitable for clinical and community settings. Third, this instrument has been validated and effectively utilized in numerous studies involving chronic pediatric condition management and caregiver outcomes (19, 20).

Before the CASAP intervention, a baseline survey was conducted by neurology nurses among the primary family caregivers of children with epilepsy who met the inclusion criteria. Specifically, data pertaining to the 6-month period immediately preceding the commencement of intervention were collected. Data were collected through hospital electronic medical records, personalized conversations with the primary family caregivers of children with epilepsy, telephonic follow-ups, and WeChat questionnaire stars. The data mainly included the total number of seizures recorded by family caregivers during that 6-month pre-intervention period, the average duration of seizures, total number of emergency visits, and results of the recent EEG examination. Primary family caregivers of children with epilepsy were required to complete a Chinese version of the Family Care Index questionnaire.

The CASAP intervention itself had a duration of six months. The post-intervention measurement was conducted at the end of this 6-month intervention period. At this time point, neurology nurses collected data for the 6-month intervention period through telephone or outpatient follow-up, either personalized or via WeChat Questionnaire Star. The post-intervention data included the total number of epileptic seizures, average duration of seizures, total number of emergency visits recorded during the intervention period, and the results of the most recent EEG examination. Primary family caregivers were required to complete the Chinese version of the Family Care Index questionnaire again.

### Data analysis

The primary strength for addressing potential confounding in this study lies in its within-subject paired design. Each child-caregiver dyad served as its own control, with pre-intervention (baseline) data compared directly to post-intervention data. This design inherently controls for all time-invariant individual characteristics. Accordingly, the statistical analysis was tailored to this paired structure. Statistical analysis of the data was conducted using the R software (version 4.3.1). The normal distribution in the measurement data were expressed as $\bar{x}$ ± s, and the paired sample *t*-test was used to compare the differences before and after the intervention. Non-normally distributed data were expressed as M (P25, P75). The Wilcoxon signed-rank sum test was used to compare differences before and after the intervention, and the Mann—Whitney *U*-test was used for comparison between groups. In addition, the number of emergency visits was classified as a binary variable (0 times, ≥1 time), and statistically described by the number of cases and percentage [*n* (%)]. The ^**^McNemar-test was used to compare differences before and after the intervention. Statistical significance was set at *P* < 0.05.

## Results

### Demographic and disease characteristics

A total of 62 children with epilepsy aged 0–18 years were included in this study, of whom 30 were boys (48%) and 32 were girls (52%); the majority of cases were aged 0–3 years (30 cases, 48%). According to the type of epilepsy, there were 20 cases (32%) of Generalized epilepsy, 20 cases (32%) of Combined Generalized & Focal epilepsy, 17 cases (28%) of Focal epilepsy, and five cases (8%) of Unknown epilepsy. Additional characteristics are listed in Table 1.

**Table 1:** Demographic and disease characteristics of the participants included in the analysis (*n* = 62).

**Characteristic**	***n* (%)**
**Age**	
Ages 0–3	30 (48%)
Ages 4–8	17 (27%)
Ages 9–11	10 (16%)
Ages 12–14	4 (7%)
Ages 15–18	1 (2%)
**Gender**	
Male	30 (48%)
Female	32 (52%)
**Epilepsy type**	
Unknown epilepsy	5 (8%)
Generalized epilepsy	20 (32%)
Focal epilepsy	17 (28%)
Combined generalized & focal epilepsy	20 (32%)
**Types of medical insurance**	
Personal (commercial) insurance	3 (5%)
Public (medical) insurance	57 (92%)
No insurance	2 (3%)
**Location**	
Rural	27 (44%)
Towns and cities	35 (56%)
**Primary caregiver**	
Parents	60 (97%)
Others	2 (3%)
**Antiepileptic drugs**	
Use	22 (36%)
Non-use	40 (64%)
**Epilepsy-related comorbidities**	
With comorbidities	25 (40%)
Without comorbidities	37 (60%)

### Outcome indicators

The outcome measures of this study included the total number of epileptic seizures within six months after the CASAP intervention, the average duration of seizures, the total number of emergency visits, the results of the recent EEG examination, and the Family Care Index questionnaire score of the main family caregivers (Table 2).

**Table 2:** Comparison of outcome variables before and after CASAP intervention.

**Outcome variable**	**Pre-intervention 6-month period: M (P25, P75)**	**Post-intervention 6-month period: M (P25, P75)**	**Statistic (z/McNemar's χ^2^)**	***P***
Total number of episodes	9 (3–169)	7 (0–20)	−2.11	0.035
Mean duration of episodes (min)	1.5 (1.0–5.4)	0.5 (0.0–1.0)	−4.93	< 0.001
Total number of emergency department visits	0 (0–1)	0 (0–0)	−2.02	0.044
Classification of emergency department visits, *n* (%)	–	–	5.03	0.042
0 time	35 (56%)	47 (76%)	–	–
≥1 time	27 (44%)	15 (24%)	–	–
Most recent EEG findings	–	–	0.00	>0.999
Abnormal	48 (77%)	49 (79%)	–	–
Normal	14 (23%)	13 (21%)	–	–
Family Functioning Scale Score (points)	5 (3–7)	7 (4–8)	2.09	0.037

CASAP, China acute seizure action plan; EEG, electroencephalogram.

#### Total number of epileptic seizures

Before the intervention with CASAP, the M (P25, P75) of the total number of episodes was nine (3–169), and seven (0–20) after the intervention (*P* < 0.05). Compared to before the intervention, although the median decreased, the degree of dispersion was still relatively large. However, the overall data range narrowed, indicating that the number of seizures in some patients decreased; however, some patients continued to have seizures.

#### Average duration of seizure

Before the intervention with CASAP, the M of the average duration of epileptic attack was 1.5 (1.0–5.4), and 0.5 (0.0–1.0) after the intervention (*P* < 0.001). Following the CASAP intervention, the average duration of seizures was shorter, and the degree of dispersion was reduced.

#### Total number of emergency visits

Before the intervention with CASAP, there were 35 patients (56%) with no emergency visits and 27 patients (44%) with ≥1 emergency visit. After CASAP intervention, the number of patients with no emergency visits increased to 47 (76%), while the number of patients with ≥1 emergency visit decreased to 15 (24%). According to McNemar's *x*^2^-test, the statistic was 5.03, and the difference was statistically significant (*P* < 0.05), suggesting that the intervention measures may have a positive effect on reducing the number of emergency visits.

#### The result of the most recent EEG examination

Before the intervention with CASAP, 48 patients (77%) had abnormal EEG, and 14 patients (23%) had normal EEG. After the intervention, 49 patients (79%) had abnormal EEG, and 13 patients (21%) had normal EEG. The changes in EEG results before and after the intervention were not significant (*P* > 0.05).

#### Score of the family function status of the primary family caregiver

Before the intervention with CASAP, the M of the total family function score of the main family caregivers was five (3–7), and after the intervention, the M (P25, P75) was seven (4–8), and the difference was statistically significant (*P* < 0.05). The Wilcoxon rank-sum test showed that the family function score after intervention was significantly higher than before intervention (*P* < 0.05).

## Discussion

The results showed that the total number of seizures in children with epilepsy within six months after CASAP intervention was lower than six months before the intervention, and the difference was statistically significant (*P* < 0.05), suggesting that CASAP may have a positive effect in controlling acute seizures in children with epilepsy. The key to analyzing the reasons may lie not only in the CASAP tool itself but also in the application of the diversified CASAP health education toolkit led by professional neurology nurses, which is implemented as a supporting measure. While this study did not directly measure health literacy or self-management, synthesizing evidence from chronic disease management literature suggests that structured educational interventions similar to CASAP often work by enhancing these intermediary factors (21–23). Studies have applied the participatory health education toolkit to the health education of patients with type 2 diabetes, and results indicate that the application of the participatory health education toolkit can significantly improve the disease management ability of patients (24). By analogy, we posit that the CASAP programme, through systematic education and skill training, likely improved caregivers' epilepsy-specific health literacy and self-management capabilities. Improvements in these intermediate outcomes may constitute a key pathway leading to the ultimate reduction in seizure recurrence.

Neurology specialist nurses applied the CASAP Health Education Toolkit to provide 20–30 min of structured personalized seizure management guidance to the primary family caregivers of children with epilepsy at each follow-up visit. They also combined videos of a standardized treatment process for acute seizures in children with epilepsy through explanations, demonstrations, and exercises. The precise delivery of the core content of the CASAP tool and comprehensive expertise of out-of-hospital seizure management skills by family caregivers were ensured. Since a single ASAP template is not suitable for all children with epilepsy and their families, the effective implementation of individualized ASAP is the key to resolving the challenges faced by families of children with epilepsy (25). This process of individualized education and empowerment is a recognized strategy for improving health literacy and fostering active self-management in chronic conditions (26, 27), which could explain the observed gains in caregiver competence and confidence.

Timely identification and standardized handling of acute seizures outside the hospital for epilepsy are at the core of preventing serious consequences, such as death, immediate complications, and cognitive-behavioral disorders (8). In the process of safe treatment outside the hospital, accurately determining the timing of transfer is a key link that directly affects the safety and treatment effects of child patients (28). The duration of epileptic seizures is a key factor in determining whether a transfer is needed. Prolonged seizures can cause irreversible damage to the brain and body organs (29). CASAP provides a basic action framework for family caregivers by following the “3S” principles (STAY, SAFE, SIDE) and the “two don'ts” principles (no pressing of limbs, no stuffing of mouths) (25, 30). The results suggested that after the CASAP intervention, the total number of emergency visits by children with epilepsy decreased (*P* < 0.05), and the average duration of seizures decreased (*P* < 0.001). Shortening the duration of the epileptic directly reflects the efficacy and timeliness of out-of-hospital emergency interventions at home under the guidance of the CASAP. We speculate that this improvement stems from enhanced health literacy, enabling caregivers to better recognize seizure severity and form improved self-management skills, allowing for quicker, more appropriate first-aid responses. Under the guidance of the CASAP, family caregivers can quickly and accurately implement safety protection and seizure management, effectively avoiding secondary injuries or seizure prolongation caused by improper intervention. A reduction in the number of emergency visits is an important indirect indicator for optimizing emergency rescue strategies, suggesting that the CASAP effectively empowers family caregivers to assess the attack situation accurately and implement initial treatment.

The active participation and collaborative ability of family caregivers directly affect the effectiveness of emergency rescue and quality of disease management. The active participation of family caregivers in the entire process of ASAP formulation and implementation is conducive to the formation of a family centered out-of-hospital seizure management system for children with epilepsy and promotes the effective implementation of personalized emergency rescue plans (16). The results of this study showed that after the CASAP intervention, the Family Care Index score of the main family caregivers of children with epilepsy was higher than before the intervention (*P* < 0.05), suggesting that CASAP can promote the improvement of family function in children with epilepsy. This improvement may be mediated by gains in health literacy and self-efficacy—core components of self-management. This might be because, by participating in the formulation and application of the CASAP, family caregivers can systematically master knowledge about epilepsy and standardized first-aid skills, be more composed and confident in the face of emergencies, and reduce panic and helplessness. Furthermore, the implementation model of CASAP has successfully transformed the primary family caregivers from passive “command recipients” in the traditional medical model into active “decision-makers and executors” in off-hospital seizure management, enhancing their control and sense of efficacy in off-hospital seizure management. This dynamic shift in role embodies the core of successful self-management and is consistent with interventions in other chronic diseases that target health literacy and patient activation to improve outcomes.

### Limitations

As a preliminary pilot study, the focus of this research was to validate the acceptability, feasibility and efficacy of the CASAP tool, without considering long-term application. Additionally, due to the small sample size, and the outcome indicators relied exclusively on the subjective reports of family caregivers, that lacked objective verification. Furthermore, this study did not measure potential mediating variables such as health literacy and self-management. Future research should incorporate validated scales to assess these constructs, which would allow for an accurate elucidation of the causal pathway through which the CASAP intervention exerts its effects. These limitations affect the generalisability of the findings, and a more comprehensive interpretation of the tool's efficacy warrants further assessment in larger, more methodologically rigorous trials.

## Conclusion

The potential efficacy of the CASAP as an out-of-hospital seizure management tool in children with epilepsy was preliminarily verified through a small-scale pilot study in the neurology center of a tertiary children's hospital in the southwest region. Although this study has certain limitations, it provides a valuable reference for improving the current out-of-hospital seizure management in children with epilepsy. In the future, it is necessary to increase the sample size, conduct prospective large-scale multicentric studies, introduce objective evaluation indicators and long-term follow-up, to provide reliable evidence for large-scale promotion and application, as well as optimisation of the CASAP tool.

## Data Availability

The original contributions presented in the study are included in the article/supplementary material, further inquiries can be directed to the corresponding author.

## References

[1] Jiang Y. To emphasize the top-level design and the timely and reasonable use of multiple methods in the therapy of children with epilepsy. Chin J Pediatr. (2020) 58:867–70. doi: 10.3760/cma.j.cn112140-20200916-00879 33120455

[2] GBD 2016 Epilepsy Collaborators. Global, regional, and national burden of epilepsy, 1990–2016: a systematic analysis for the global burden of disease study 2016. Lancet Neurol. (2019)18:357–75. doi: 10.1016/S1474-4422(19)30120-6 30773428 PMC6416168

[3] Yang W Cui Y Ma Y Shuai J Yan Y. Trends in disease burden of epilepsy among children and adolescents in China from 1990 to 2019. Chin J Health Stat. (2024) 41:274–76

[4] Xiao B Long H. The present status and prospect of antiepileptic drugs. Chin J Neurol. (2021) 54:5–8. doi: 10.3760/cma.j.cn113694-20201012-00776

[5] Tu X. Epidemiological study of epilepsy. J Brain Nerv Dis. (2017) 25:522–29.

[6] Ding D Zhou D Sander J Wang W Li S Hong Z. Epilepsy in China: major progress in the past two decades. Lancet Neurol. (2021) 20:316–26. doi: 10.1016/S1474-4422(21)00023-5 33743240

[7] Xu T Wu J Liu J Zhao Y. Analysis of medical resource utilization and direct medical burden among epilepsy patients covered by urban employee basic medical insurance in Tianjin. Chin J Health Stat. (2016) 33:1039–42.

[8] Cui C Zheng X Li S Chen W Zeng J. Application progress and prospect of acute seizure action plans for children. Chin Nurs Manag. (2022) 22:1560–5.

[9] Kaushik JS Kadwa RA Sahu JK Sharma S Mittal R. Association of child neurology (AOCN)-Indian epilepsy society (IES) SOLACE Expert Group. Association of child neurology-Indian epilepsy society consensus document on social and legal aspects of childhood epilepsy (SOLACE). Indian J Pediatr. (2019) 86:599–607. doi: 10.1007/s12098-019-02927-2 30945236

[10] Besag FM. The department of health action plan “improving services for people with epilepsy”: a significant advance or only a first step? Seizure. (2004) 13:553–64. doi: 10.1016/j.seizure.2004.01.005 15519915

[11] Albert DVF Moreland JJ Salvator A Moore-Clingenpeel M Haridas B Cole JW . Seizure action plans for pediatric patients with epilepsy: a randomized controlled trial. J Child Neurol. (2019) 34:666–73. doi: 10.1177/0883073819846810 31156013

[12] Herman ST Detyniecki K O'Hara K Penovich P Rao VR Tatum W . Written seizure action plans for adult patients with epilepsy: distilling insights from emergency action plans for other chronic conditions. Epilepsy Behav. (2023) 140:109002. doi: 10.1016/j.yebeh.2022.109002 36822041

[13] Buchhalter J Shafer PO Buelow JM French JA Gilchrist B Hirsch LJ . Preferred practices for rescue treatment of seizure clusters: a consen-sus-driven, multi-stakeholder approach. Epilepsy Behav. (2021) 117:107836. doi: 10.1016/j.yebeh.2021.107836 33640567

[14] Patel AD Becker DA. Introduction to use of an acute seizure action plan for seizure clusters and guidance for implementation. Epilepsia. (2022) 63:S25–33. doi: 10.1111/epi.17344 35999175

[15] Penovich PE Buelow J Steinberg K Sirven J Wheless J. Burden of seiz-ure clusters on patients with epilepsy and caregivers: survey of patient, caregiver, and clinician perspectives. Neurologist. (2017) 22:207–14. doi: 10.1097/NRL.0000000000000140 29095321

[16] Guo S Yan L Zheng X Cui C. Summary of the best evidence for out-of-hospital management of acute seizures in children. J Nurses Train. (2024) 39:1754–9.

[17] Cui C Li S Chen W Zhou H Zheng X. Chinese families' knowledge, attitudes, and practices regarding seizure management for children with epilepsy: a mixed-methods study. Front Public Health. (2023) 11:1081720. doi: 10.3389/fpubh.2023.1081720 37255754 PMC10225546

[18] Smilkstein G Ashworth C Montano D. Validity and reliability of the family APGAR as a test of family function. J Fam Pract. (1982) 15:303–11. 7097168

[19] Zhang M Zhang W Liu Y Wu M Zhou J Mao Z. Relationship between family function, anxiety, and quality of life for older adults with hypertension in low-income communities. Int J Hypertens. (2021) 2021:5547190. doi: 10.1155/2021/5547190 34616569 PMC8490058

[20] Huang Y Liu Y Wang Y Liu D. Family function fully mediates the relationship between social support and perinatal depression in rural Southwest China. BMC Psychiatry. (2021) 21:151. doi: 10.1186/s12888-021-03155-9 33711987 PMC7953569

[21] van der Gaag M Heijmans M Valli C Orrego C Ballester M Rademakers J. Self-management interventions for chronically ill patients with limited health literacy: A descriptive analysis. Chronic Illn. (2024) 20:578–604. doi: 10.1177/17423953231181410 37312500

[22] Cabezas MF Nazar G Ranchor AV Annema C. The effect of health literacy interventions on self-management in chronic diseases: a systematic review. Ann Behav Med. (2025) 59:kaaf073. doi: 10.1093/abm/kaaf073 41176327 PMC12579557

[23] Leviton A Patel AD Loddenkemper T. Self-management education for children with epilepsy and their caregivers. A scoping review Epilepsy Behav. (2023) 144:109232. doi: 10.1016/j.yebeh.2023.109232 37196451

[24] Li A Wang L Wang Y Dong H Xu H Wang X. Application of a participatory health education toolkit in patients with type 2 diabetes mellitus. Chin Nurs Manag. (2024) 24:787–92.

[25] Penovich P Glauser T Becker D Patel AD Sirven J Long L . Rec-ommendations for development of acute seizure action plans (ASAPs) from an expert panel. Epilepsy Behav. (2021) 123:108264. doi: 10.1016/j.yebeh.2021.108264 34482230

[26] Downie S Shnaigat M Hosseinzadeh H. Effectiveness of health literacy- and patient activation-targeted interventions on chronic disease self-management outcomes in outpatient settings: a systematic review. Aust J Prim Health. (2022) 28:83–96. doi: 10.1071/PY21176 35131029

[27] Mackey LM Doody C Werner EL Fullen B. Self-management skills in chronic disease management: what role does health literacy have? Med Decis Making. (2016) 36:741–59. doi: 10.1177/0272989X16638330 27053527

[28] Glauser T Shinnar S Gloss D Alldredge B Arya R Bainbridge J . Evidence-based guideline: treatment of convulsive status epilepticus in children and adults: report of the guideline committee of the american epilepsy society. Epilepsy Curr. (2016) 16:48–61. doi: 10.5698/1535-7597-16.1.48 26900382 PMC4749120

[29] Au CC Branco RG Tasker RC. Management protocols for status epilepticus in the pediatric emergency room: systematic review article. J Pediatr (Rio J). (2017) 93:84–94. doi: 10.1016/j.jped.2017.08.004 28941387

[30] McKenzie KC Hahn CD Friedman JN. Emergency management of the paediatric patient with convulsive status epilepticus. Paediatr Child Health. (2021) 26:50–66. doi: 10.1093/pch/pxaa127 33552322 PMC7850284

